# The Twin Cycle Hypothesis of type 2 diabetes aetiology: From concept to national NHS programme

**DOI:** 10.1113/EP092009

**Published:** 2025-02-03

**Authors:** Roy Taylor

**Affiliations:** ^1^ Magnetic Resonance Centre, Translational and Clinical Research Institute Newcastle University Newcastle upon Tyne UK

**Keywords:** clinical applications, dietary weight loss, Twin Cycle Hypothesis, type‐2 diabetes

## Abstract

The development of magnetic resonance methods for quantifying intra‐organ metabolites has permitted advances in the understanding of fasting and post‐prandial carbohydrate and lipid handling in people with and without type 2 diabetes. Insulin resistance in the liver was shown to be related to excess intra‐organ fat and was able to be returned to normal by weight loss. The practical effect of having muscle insulin sensitivity in the lower part of the wide normal range resulted in the obligatory shunting of carbohydrates via *de novo* lipogenesis into saturated fat. These observations provided the basis for the Twin Cycle Hypothesis of the aetiology of type 2 diabetes. Subsequent studies on people with type 2 diabetes confirmed the postulated pathophysiological abnormalities and demonstrated their reversibility by dietary weight loss of 10–15 kg. Overall, the fundamental understanding of the mechanisms causing type 2 diabetes has bridged physiological and clinical perspectives. Large population‐based randomised controlled trials confirmed the practical clinical application of the method of achieving substantial weight loss, and an NHS programme is now in place offering potential remission to people within 6 years of diagnosis.

## Abstract (highlights)

- **What is the topic of this review?**
  Pathophysiology of type 2 diabetes and reversal to normal physiology by dietary weight loss.
- **What advances does it highlight?**
  Testing the Twin Cycle Hypothesis has shown that type 2 diabetes is a potentially reversible metabolic state. The necessary 10–15% weight loss brings about the normalisation of hepatic triglyceride content, hepatic triglyceride export and β‐cell fat exposure, with β‐cell re‐differentiation and return of glucose responsiveness.

## 1 BACKGROUND

The aetiology of type 2 diabetes has been uncertain since its recognition as a separate condition from type 1 diabetes over half a century ago. Because of the different clinical courses in different individuals and the different physical characteristics of those developing type 2 diabetes, it has become widely believed that the aetiology is heterogeneous (Defronzo, 2009; Schon et al., 2024). Theoretical concepts lacking solid evidence, for instance concerning the microbiome, inflammation and β‐cell apoptosis, have contributed to this belief. Furthermore, investigation of the aetiology of type 2 diabetes usually considers separate mechanisms affecting muscle, liver and pancreas. However, the physiology of food handling in the normal state and how this may become disordered in metabolic disease has been little considered. Advances in magnetic resonance spectroscopy have allowed observation of the dynamic changes inside the major metabolic organs (Petersen et al., 2005; Ravikumar et al., 2008; Shulman et al., 1990; Singhal et al., 2002; Taylor et al., 1992). Consequently, a series of studies was undertaken leading to an understanding of the aetiology of type 2 diabetes and the development of methods to achieve a return to metabolic health.

## 2 STUDIES ON SKELETAL MUSCLE

The advent of infinitely repeatable, non‐invasive measurement of glycogen inside metabolic organs by magnetic resonance spectroscopy allowed measurement of the time course and extent of storage of meal carbohydrates as muscle glycogen. After a lag phase when glucose oxidation predominated, storage proceeded rapidly and peaked at 5 h after eating (Taylor et al., 1993). At that time, 36% of the meal carbohydrate was stored as muscle glycogen in people with normal muscle insulin sensitivity. Notably, this declined after that peak most probably by means of lactate export to the liver. The resulting carbon utilisation by gluconeogenesis contributes to the ongoing hepatic glucose output. The phasic time course of operation of what is known as the Cori cycle had not previously been appreciated.

The earliest detectable signal of the likelihood of developing type 2 diabetes is relatively low insulin sensitivity in muscle (Lillioja et al., 1988). Although described as ‘insulin resistance’, the point is often overlooked that there is a wide distribution of this parameter in the normal population which encompasses the distribution seen in type 2 diabetes (Taylor, 2012). The latter is merely skewed towards lower sensitivity with no cut‐off of ‘normal’ or ‘abnormal’, and this characteristic appears to be largely genetic (Warram et al. 1990; Martin et al., 1992; Petersen et al., 2007) and is detectable in early life in those most likely to develop type 2 diabetes (Defronzo et al., 2009). In such people, only 8% of the ingested carbohydrate becomes stored as muscle glycogen at the peak of 5 h (Carey et al., 2003). During the 24h cycle when three meals are eaten, muscle glycogen levels rise sequentially and then fall overnight, contributing to the necessary continuous hepatic glucose production. The stark contrast between people with average insulin sensitivity and those with the degree typically seen in type 2 diabetes is underscored by the observation of considerable diurnal flux of muscle glycogen in the former but none in the latter (Macauley et al., 2015).

These observations are critical to understanding both the pathophysiology of type 2 diabetes and the contributary factors for coronary heart disease. People with low sensitivity to insulin in muscle are less able to store food‐derived glucose as glycogen, and this glucose has to be dealt with by the only other available pathway, of *de novo* lipogenesis (Macauley et al., 2015; Petersen et al., 2007; Rabol et al., 2011). The sole product of lipogenesis is palmitic acid, a 16‐carbon saturated fatty acid. Hence after every meal, every day the body is exposed to excess saturated fat, the specific type most inhibitory of β‐cell function (Morgan et al., 2008) and that most associated with coronary heart disease.

## 3 STUDIES ON LIVER

Around 20 years ago the association between high levels of intra‐hepatic fat and type 2 diabetes began to be recognised (Petersen et al., 2005; Singhal et al., 2002; Yki‐Jarvinen, 2005). Demonstration that the level of liver fat was directly proportional to the degree of insulin resistance of the liver was important in directing physiological investigation of these parameters (Singhal et al., 2002). Moderate dietary weight loss brings about both fall to normal of liver fat content and hepatic insulin resistance (Petersen et al., 2005; Yki‐Jarvinen, 2005). The rapidity of the resulting fall in fasting plasma glucose was first demonstrated after bariatric surgery but the observation was misinterpreted, being thought to be an effect of the post‐prandial spike of glucagon‐like peptide 1 (GLP‐1) (Guidone et al., 2006). This was untenable simply because no food was taken orally in the 7 days after the bilio‐pancreatic diversion surgery and fasting plasma glucose was normal by 7 days post‐operatively. This necessity to allow post‐operative healing of anastomoses after the extensive bypass procedure is described in the Methods of Guidone et al. (2006) but was not factored into interpretation of the data. The participants had a mean BMI of 55 kg/m^2^ and would require at least 2800 kcal/day for basal metabolism alone and more to support the extra physical energy to move a very heavy body. Even though intravenous feeding aimed to provide 1000 kcal/day, the energy deficit during the immediate post‐operative period readily explained the rapid normalisation of hepatic insulin sensitivity. Remarkably, the misconception about an incretin‐based mechanism of the rapid decrease in fasting plasma glucose has persisted despite clear evidence to the contrary (Eriksson et al., 2024; Steven et al., 2015, Steven, Hollingsworth, Small, Woodcock, Pucci, Aribisala, Al‐Mrabeh, Batterham, 2016; Yoshino et al., 2020).

## 4 KNOWLEDGE OF β‐CELL FUNCTION

Taken together, these observations offered an explanation for the very gradual increase in fasting plasma glucose during the development of type 2 diabetes and the rapid return to normal following a short‐term but major calorie deficit. This did not explain the development of β‐cell dysfunction in response to a post‐prandial rise in plasma glucose, but insight was provided serendipitously by observation of a personal patient with type 2 diabetes. This person was insistent upon achieving complete relief from type 2 diabetes and was advised that fasting glucose could almost certainly be normalised by returning to the same body weight as in early adult life. The weight loss was achieved and not only did fasting glucose return to normal, but the oral glucose tolerance curve also normalised 3 months after commencing the significant decrease in food intake. It was becoming apparent that the abnormalities of type 2 diabetes affecting the liver and pancreas were linked. Chronic provision of saturated fat to perifused rodent β‐cells had been observed to cause loss of first‐phase insulin response over a decade earlier (Lee et al., 1994). The Twin Cycle Hypothesis was postulated as a result of combining the experimental and clinical data. I first published this in this paper which was invited by the Editor of Diabetologia following the first oral presentation of my hypothesis to the Diabetes UK Annual Scientific meeting in (Taylor, 2008).

## 5 TWIN CYCLE HYPOTHESIS

**FIGURE 1:**
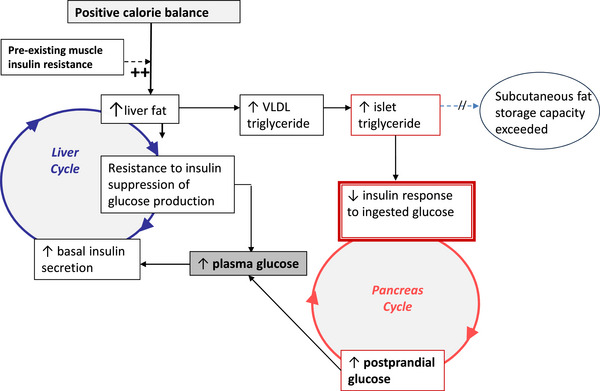
The Twin Cycle Hypothesis. Each of the steps is described in the text. Figure reproduced with permission from Taylor (2008).

Chronic positive calorie balance promotes an increase in fat content at all sites including the liver. However, the excess carbohydrate which cannot be stored as glycogen in skeletal muscle has to be handled by *de novo* lipogenesis, a process which particularly promotes fat accumulation in the liver. Higher levels of fasting insulin will stimulate the process, naturally to a greater extent in those individuals with a degree of insulin resistance. The increased liver fat in turn will cause relative resistance to the minute‐by‐minute insulin suppression of hepatic glucose production. Over many years, fasting plasma glucose levels will increase, causing a further increase in basal insulin secretion rates to maintain euglycaemia (Taylor, 2008). The consequent hyperinsulinaemia will enhance the conversion of excess calories into liver fat. A vicious cycle of hyperinsulinaemia and blunted suppression of hepatic glucose production becomes established. Fatty liver leads to increased export into the circulation of very‐low‐density lipoprotein (VLDL) triglyceride (TG) (and hence excess plasma triglyceride) (Adiels et al., 2006), which will increase fat delivery to all tissues including the islets. Lipoprotein lipase is present in the capillaries of the islets. The rise in fasting and post‐prandial plasma glucose will further increase the rate of VLDL‐TG production (Adiels et al., 2006), ensuring excess fatty acid availability in the pancreatic islet, which would impair the acute insulin secretion following meals, and eventually post‐prandial hyperglycaemia will supervene. Hyperglycaemia will further increase insulin secretion rates, with knock‐on effects on hepatic lipogenesis, spinning the liver cycle faster and hence the same for the pancreas cycle. Eventually, the fatty acid and glucose inhibitory effects on the islets reach a level causing failure of β‐cell response to an increase in plasma glucose and a relatively sudden onset of clinical diabetes. The cycle is shown diagrammatically in Figure 1.

### 5.1 Testing the Twin Cycle Hypothesis

The main purpose of laying out a detailed hypothesis explaining physiological and pathophysiological events is to permit construction of an experimental design which can disprove the hypothesis. If the resulting observations are compatible with the hypothesis then it may be correct. The Twin Cycle Hypothesis could easily be disproven if people with type 2 diabetes were exposed to a calorie deficit similar to that observed after bariatric surgery and plasma glucose levels did not return to normal. The extent of predicted changes in plasma glucose, insulin and triglyceride were so great that the prior power calculation indicated the need for only 12 participants. The CounterPOINT (Counteracting Pancreatic inhibitiOn of Insulin secretion by Triglyceride) study aimed to test the hypothesis by measuring changes during an 8 week period of 800 kcal/day in not only the main plasma analytes but also the underlying processes and intra‐organ fat content (Lim et al., 2011). The results were striking.

**FIGURE 2:**
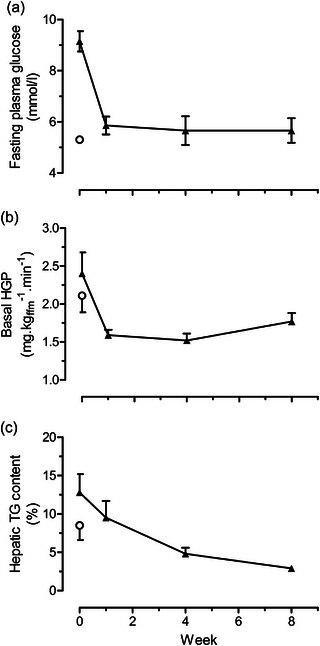
Effect of 8 weeks of dietary intervention on (a) plasma glucose, (b) HGP and (c) hepatic TG content for subjects with type 2 diabetes (filled triangles). Open circles indicate mean of the weight‐matched normoglycaemic control group. Data are shown as means ± SE. Figure reproduced with permission from Lim et al. (2011). HGP, hepatic glucose production; TG, triacylglycerol.

**FIGURE 3:**
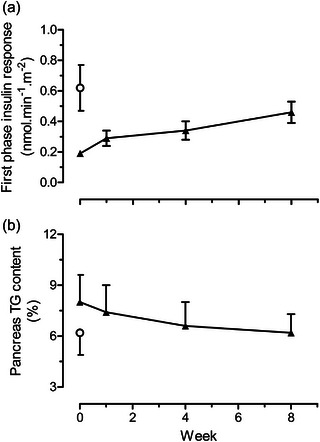
(a) Change in first phase insulin response and (b) change in pancreas TG content during the 8‐week dietary intervention in subjects with type 2 diabnetes (filled triangles). Open circles indicate mean of the weight‐matched normoglycaemic control group. Data are shown as mean ± SE. Figure reproduced with permission from Lim et al., 2011. TG, triacylglycerol.

Fasting plasma glucose and insulin returned to normal within 1 week, associated with major decreases in liver fat content and hepatic glucose production (Figure 2). High statistical significance was achieved for all measurements. A gradual decrease in intra‐pancreatic fat content was matched by a steady return towards normal in the first phase of insulin response (Figure 3). The latter was particularly notable as it had previously been believed that the slow decline of β‐cell function was irreversible. Notably, the return to the non‐diabetic state was achieved with no change in the degree of muscle insulin resistance, which is largely genetically determined, unlike liver insulin resistance.

The effect size of all the predicted changes in returning to normal made it probable that the hypothesis under test was not only consistent with observations but was also correct. The postulated liver and pancreas cycles were in effect put into reverse, with reversal to non‐diabetic plasma glucose levels. Media coverage of the research resulted in a large influx of emails from people with diabetes and it was possible to collate their observations (Steven et al., 2013). That this represented a true change of state rather than a temporary response to calorie restriction was demonstrated in the second major study. CounterBALANCE was designed to examine this question, in parallel with a second question of whether type 2 diabetes of any duration could be reversed by substantial weight loss. The CounterPOINT participants had been selected as having type 2 diabetes diagnosed within the previous 4 years whereas CounterBALANCE recruited people with any type 2 diabetes duration. Again, clear data were obtained (Steven et al., 2015, Steven, Hollingsworth, Al‐Mrabeh, et al. 2016). During 6 months of follow‐up with avoidance of weight regain, those who achieved remission exhibited continuing normality of all parameters. Remission was demonstrated for the first time. There was a clear relationship between the duration of known type 2 diabetes and the ability to return to the non‐diabetic state with a steady decline in the latter.

CounterBALANCE also provided useful insight into why some people did not achieve remission despite adequate weight loss. Everyone achieved normalisation of the liver cycle, with a return to normal levels of intrahepatic fat and rates of VLDL‐TG export. However, despite normalisation of plasma triglyceride levels and intra‐pancreatic fat content, not everyone could achieve restoration of β‐cell function. It appears that permanent loss of ability to return to specialised function occurs if exposure to high triglyceride and raised plasma glucose is prolonged. If diabetes had been diagnosed within 4 years, 87% returned to non‐diabetic control compared with 50% if the diagnosis had been made more than 8 years ago. All subsequent studies and consequently the new NHS national programme were therefore limited to those within 6 years of diagnosis of type 2 diabetes even though this is not an absolute cut‐off. Genetic factors concerning the resilience of the β‐cell to fat‐induced suppression appear likely to explain individual responses, from early loss of response to the occasionally observed recovery despite diabetes over 20 years.

Comparison of people with or without type 2 diabetes studied during dietary‐ or bariatric surgery‐induced weight loss in the Pancreas study confirmed that identical pathophysiological changes explained the return of normoglycaemia and that these occurred whether or not post‐surgical incretin hormones spiked after eating (Steven, Hollingsworth, Small, Woodcock, Pucci, Aribisala, Al‐Mrabeh, Batterham, et al., 2016).

Together, the above studies paved the way for a randomised controlled trial of substantial weight loss using the same effective dietary method. The detailed studies allowed separation of the independent changes of insulin sensitivity in the liver and muscle [Al‐Mrabeh et al 2020 and Taylor et al (2018)], explaining much of the previous confusion about whole body estimates which did not distinguish between the very different components.

## 6 THE DIABETES REMISSION CLINICAL TRIAL (DiRECT)

DiRECT was conducted in Primary Care by providing training in effective weight loss for the NHS nurses or dietitians who delivered routine diabetes care. Two hundred and ninety‐eight people were randomised equally to have the best management by current guidelines (National Institute for Clinical Excellence and Scocttish Intercollegiate Network) or to have medications stopped and to commence the rapid weight loss programme (Leslie et al., 2016; Taylor et al., 2017). The mean weight loss in the intervention group at 12 months was 10 kg (analysed on an intention‐to‐treat basis) with diabetes remission, off all anti‐diabetes drugs, in 46% (Lean et al., 2018). The mean weight loss in the control group at 12 months was 1 kg and 4% were in remission. At 2 years, 36% of the weight loss group were still in drug‐free remission (Lean et al., 2019). The study provided the opportunity to extend the pathophysiological observations.

Over the first 12 months the functional β‐cell mass, assessed as insulin secretory rate during maximal stimulation, gradually increased to that of a matched non‐diabetic control group (Zhyzhneuskaya et al., 2020). Clearly, there had been no β‐cell death or apoptosis to account for the inadequate insulin secretion previously quantified, as no measurable β‐cell regeneration in human adults has been recorded. The observed β‐cell behaviour was consistent with dedifferentiation of these specialised cells under the metabolic stress of excess local fatty acid provision via lipoprotein lipase activity on triglyceride in the islet capillary network (Accili, 2018; Cinti et al., 2016; Talchai et al., 2012; Taylor et al., 2018). Also, in a subgroup of 13 people who gained excessive weight and lost their initial remission of diabetes, it was possible to observe the time course of the changes predicted by the Twin Cycle Hypothesis. Liver fat content increased, plasma VLDL‐TG increased, palmitic acid content of VLDL‐TG increased, pancreas fat content increased, and eventually insulin secretion in response to a glucose load diminished back to the original low level (Al‐Mrabeh et al., 2020). In contrast, if excess weight regain was avoided, all the underlying pathophysiological processes remained normal or close to normal and β‐cell function remained stable.

## 7 CONFUSION ABOUT OBESITY

Type 2 diabetes is frequently described as a ‘disease of obesity’ (NICE, 2022; Targher et al., 2021). However, only approximately 50% of people have a BMI above 30 kg/m^2^ at diagnosis and 16% have a normal BMI as defined (Taylor & Holman, 2015; UKPDS, 1991). Indeed, when the background prevalence of obesity was only 7% in the 1980s, only 25% of people developing type 2 diabetes had a BMI above 30 kg/m^2^ and 36% had a normal BMI. At that time there appeared to be no link or only a tenuous link between obesity and type 2 diabetes (Jarrett et al., 1979; Taylor, 1989). Currently, it is often overlooked that BMI was developed as a metric for populations, and the measurement does not reflect the risk for an individual. Furthermore, the studies described above were conducted in people of BMI 27–45 kg/m^2^, and 15 kg weight loss produced identical changes whether an individual's BMI changed from 45 to 42 or 27 to 24 kg/m^2^ (Al‐Mrabeh et al., 2020; Lim et al., 2011; Steven, Hollingsworth, Al‐Mrabeh, et al., 2016, Steven, Hollingsworth, Small, Woodcock, Pucci, Aribisala, Al‐Mrabeh, Daly, et al., 2016; Taylor et al., 2019; Zhyzhneuskaya et al., 2020). This led to the development of the Personal Fat Threshold Hypothesis which postulated that type 2 diabetes was precipitated by an individual accumulating more fat inside the liver and pancreas than they personally could tolerate (Taylor & Holman, 2015).

This hypothesis was tested in the ReTUNE (Reversal of Type 2 diabetes Upon Normalization of Energy intake in non‐obese people) study. In a cohort of people with BMI 21–27 kg/m^2^, 70% achieved remission of diabetes at a median weight loss of 6.5 kg with the anticipated wide range of 5.5–10.2% weight loss (Taylor et al., 2023). At baseline, the pathophysiological abnormalities were the same as those of heavier people and returned to normal with weight loss in the same way.

## 8 IMPACT OF APPLYING PHYSIOLOGICAL METHODOLOGY

The possibility of repeated non‐invasive measurement of intra‐organ metabolites, coupled with standard physiological methods, has permitted rigorous testing of prior hypotheses in human type 2 diabetes. It is now clear that type 2 diabetes is a condition of homogeneous aetiology occurring in genetically heterogeneous individuals (Taylor, 2024). Previous assumptions of heterogenous causes, often invoking mechanisms lacking a plausible physiological basis, can now be set aside. But of great relevance to people who develop this condition, there is now a potential route back to full health, the number one priority of people with type 2 diabetes (Oliver & Holt, 2020; Taylor & Barnes, 2018). Before this understanding, everyone diagnosed with type 2 diabetes was informed that they had a life‐long, inevitably progressive disease with serious life‐shortening complications; this is a highly significant advance.

Unsurprisingly, polygenetic differences between ethnic groups result in some clinical differences, for instance, in BMI at presentation of type 2 diabetes. However, all populations tested show the same response to substantial weight loss (Bynoe et al., 2019; Chrakraborty et al., 2024; Taheri et al., 2020) with no suggestion of a difference in the pathophysiology of the disease.

The NHS England's Type 2 Diabetes Path to Remission Programme initially ran as a limited pilot project but its success led to a national roll‐out completed in 2024. In the first year, this population‐based programme achieved 10.2 kg weight loss and 36% with an HbA1c in the non‐diabetic range (Valabhji et al., 2024). Research based soundly upon physiological principles has led to potentially life‐changing treatment for people with type 2 diabetes.

## AUTHOR CONTRIBUTIONS

Sole author.

## CONFLICT OF INTEREST

RT is an advisor for Fast800, and is author of the book, Life Without Diabetes (all royalties to Diabetes UK).

## FUNDING INFORMATION

No funding was received for this work.
